# Improving Special Ability Performance of Badminton Players through a Visual Reaction Training System

**DOI:** 10.3390/healthcare10081454

**Published:** 2022-08-02

**Authors:** Kuei-Pin Kuo, Chun-Chin Liao, Chun-Chieh Kao

**Affiliations:** 1 Office of Physical Education, National Ping-Tung University of Science and Technology, 1 Shuefu Road, Neipu, Pingtung 91201, Taiwan; kweibin0422@gmail.com; 2 Office of Physical Education, Ming Chuan University, 5 De Ming Road, Gui Shan District, Taoyuan City 333, Taiwan

**Keywords:** footwork, reaction time, agility, performance

## Abstract

This study investigates the effects of a visual reaction training system (VRTS) in improving the footwork of badminton players. The participants comprised 20 high school male badminton players (mean age, 17.83 ± 1.57 years; mean height, 171.4 ± 11.52 cm; mean weight, 58.76 ± 9.32 kg) who first underwent a badminton footwork agility training program and subsequently, a fixed or random six-point footwork test and an agility *t*-test. A one-way repeated-measures analysis of variance with Bonferroni correction was performed to identify differences in terms of response time, movement time, and total shift time. The results measured at midtest and posttest after the training intervention revealed significant improvements in reaction (*p* ≤ 0.01) and movement (*p* ≤ 0.05) time for the fixed six-point footwork test (*p* ≤ 0.01). The total time results for the fixed or random six-point footwork test and agility *t*-test at midtest and posttest after the training intervention revealed significant improvement (*p* ≤ 0.05). Badminton footwork agility training conducted through the VRTS enhances the ability and agility of badminton players. Therefore, researchers and coaches should evaluate the footwork of badminton players by precisely measuring and quantify their ability.

## 1. Introduction

The initial speed of a shuttlecock following a badminton smash is faster relative to similar movements used in other sports. In international badminton tournaments, the fastest speeds ever recorded for singles and doubles matches are 417 and 426 km/h, indicating that badminton is a fast-paced sports in which players must react to each hit with rapid movements that require agility and strategy. Numerous studies have investigated the footwork and movements of badminton players, and their findings have been combined with sports science and technology in recent years and widely applied in badminton training. In one study, accelerators were used to obtain the step-related data of players during step training, and a quantitative analysis of average and maximum speeds was performed to effectively assess the speed and state of their movements to adequately improve their agility and overall athletic performance [1,2,3,4]. Badminton involves shots that require speed and explosive strength combined with agile footwork and movements that are applied tactically, and it has become a popular racket sport that is extensively developed worldwide [5].

Research has revealed a significant correlation between athletic performance and the agility of footwork movements [1,2,3,4,6]. Traditionally, agility was simply defined as the speed with directional changes [7]. Currently, agility is considered an open skill, and it was recently defined as a change in velocity or direction in response to a stimulus that cannot be preplanned [8]. Agility is defined as a rapid whole-body movement with a change of direction or speed in response to a stimulus. However, traditional agility tests do not address this definition and are preplanned with no stimulus. Compared to traditional agility testing, reactive agility testing is an effective and reliable agility testing method. Reactive agility tests can also be used as training exercises to improve skills by using sport-specific stimuli that preplanned agility exercises may not have. Players who can perform rapid movements are likely to dominate in badminton tournaments [9,10]. Therefore, the footwork of badminton players during rapid movements is a crucial basis for defining agility [11,12,13]. Although agility is a key ability required in sports, its definition has remained divergent until recently. Young et al. proposed that agility involves cognitive skills, physical qualities, and technical aspects. Cognitive skills comprise the time taken by an individual to make judgments and decisions as well as the reaction time of that individual [14]. Physical qualities refer to the linear sprint velocity of an individual (depending on the muscular, explosive, and reactive strength of their leg muscles) and their core muscular strength. Technical aspects comprise two indicators, namely, cognitive skills and physical qualities, which are applied strategically to perform sports techniques [15].

A badminton court comprises two halves that each measure 6.7 m in length and 6.1 m in width. The six primary directions of movement on the court are leftward and rightward in the forecourt, midcourt, and rear court. The strategies for reacting to an opponent and executing footwork vary depending on the movements made in these six directions, which include the cross-step in the forecourt and rear court and the lunge in the midcourt. Badminton players who are able to complete their footwork faster show greater average and maximum acceleration. Players deploy movement strategies with the center of the court as the starting and ending points for shifting and returning. Steps, lunges, and other types of footwork movements are performed to receive the shuttlecock and to return to a defensive position after each attack and defense as soon as possible to correctly per-form efficient (direction and distance) and high-quality (strength and intensity) shots. Immediately returning to the central position after performing a shot and following up with another shot is an efficient technique that increases a badminton player’s probability of winning [1,2].

In recent years, numerous studies have analyzed the movements of badminton player by employing kinesiological research methods, and they revealed that footwork during badminton movements is a key indicator of a badminton player’s athletic performance and a basis for evaluating their injury risk. Findings in the literature have also indicated that a higher percentage of the injuries sustained in badminton occur in the lower limbs than in other parts of the body [16,17]. Therefore, an appropriate examination of the quantitative data on agile reactions (of badminton players) can enable researchers to understand the variability of a player’s overall trend, and overtraining and an unnecessary training load can be avoided by referring to these data and focusing on incidence statistics. Badminton movements are characterized by rapid changes in direction, which are required to achieve tournament-winning performance. A study investigated the plantar pressure variations in players with varying skill levels when they were performing lunges for net shots. Its results revealed that plantar pressure was distributed across the inner side of the feet among skilled players and across the outer side of the feet among average players; they also indicated that the difference in the distribution of pressure affected the motions of the players’ lower limb joints [18].

Lower limb joint loading affects the athletic performance of badminton players. Studies have revealed that incorrect footwork movements have a negative effect on the state of lower limb joints and cause injuries [19,20,21]. For inappropriate footwork movements involving lunges, the body compensates by shifting the trunk inclination angle of the lower limb joints, which in turn affects the efficiency of shots and causes injuries [19,20,22]. In recent years, neuromuscular theories and research results have been widely applied in various types of sports training to enhance muscular strength while correctly and effectively reducing the correlation between injury risk and muscle-controlling nerves [23,24]. Therefore, the enhancement of footwork and movement is an essential training component for badminton players. The agility *t*-test proposed by the National Strength and Conditioning Association of the United States is an effective method for measuring speed, explosive strength, and agility [25].

In recent years, the focus has shifted to training methods that are based on simulation of competition and the accurate monitoring of training outcomes through the processing of training results using quantitative methods (e.g., data collection). At present, few sport-specific scientific electronic devices with both training and testing features have been developed. Scientific quantitative training methods allow for the specific and effective modification and improvement of training programs for players with respect to their movement characteristics, advantages, and disadvantages. Numerous studies have used biomechanical methods to explore footwork movements. Although three-dimensional biomechanical motion capture systems enable researchers to effectively measure parameters of kinematics, dynamics, and athletic performance in the context of human activities, data collection is subject to environmental constraints that prevent the use of large research samples. The present study aimed to conduct intervention training and examine its outcomes with a self-developed visual reaction training system (VRTS) to test the system’s effectiveness in routine badminton training. The training system was designed to provide coaches and players with various types of quantitative data on badminton agility and specific skills, allowing them to clearly modify their subsequent training programs based on the collected data and results and to improve the tournament performance of players.

## 2. Methods

### 2.1. Participants

This study assessed the agility and specific abilities of high school badminton players after they underwent 12 weeks of specific physical training that was based on the VRTS. Twenty male high school badminton players who received regular training were recruited as the participants of the present study; the 20 participants were all members of local high school badminton teams (mean age = 17.83 ± 1.57 years; mean height = 171.4 ± 11.52 cm; mean weight = 58.76 ± 9.32 kg; the right hand is the dominant hand for all participants). To prevent research participant effects from affecting the research results of the present study during the intervention phase, participants were only included if they (1) underwent at least 6 h of specialized badminton training spread across at least three sessions per week, (2) did not participate in any neuromuscular stimulation program during the first 6 months of training, (3) had no history of neuromuscular injury in the bones of their lower extremity within 1 year, and (4) understood the experimental procedure and provided informed consent (i.e., the participants’ and their guardians’ consent). Students were directly excluded if they had a relationship with the researcher. The present study was approved by the institutional review board of Antai Tian-Sheng Memorial Hospital, Antai Medical Care Cooperation (IRB approval number: 19-034-B, Pingtung, Taiwan).

### 2.2. Research Tools

#### VRTS

In accordance with the methodology of another study [3], the VRTS used in the present study mainly comprised a programmable logic controller and a human–machine interface. The RS232 communication protocol was used for data transmission, image conversion was conducted through the HMI, and a sequential function chart was used to write the internal syntax. The hardware devices comprised an infrared sensor module, light-emitting diode (LED) lights equipped with multicore control cables, and wireless I/O modules with a maximum transmission range of 100 m. Radio signals were transmitted through globally shared frequency bands between 2400 and 2480 MHz to send various types of data (e.g., trend analysis charts and time parameters) as feedback for coaches and players. The devices were tested using interclass correlation coefficients (ICCs) and demonstrated to exhibit high reliability and validity (ICC = 0.95) [3].

## 3. Research Procedures

**Figure 1:**
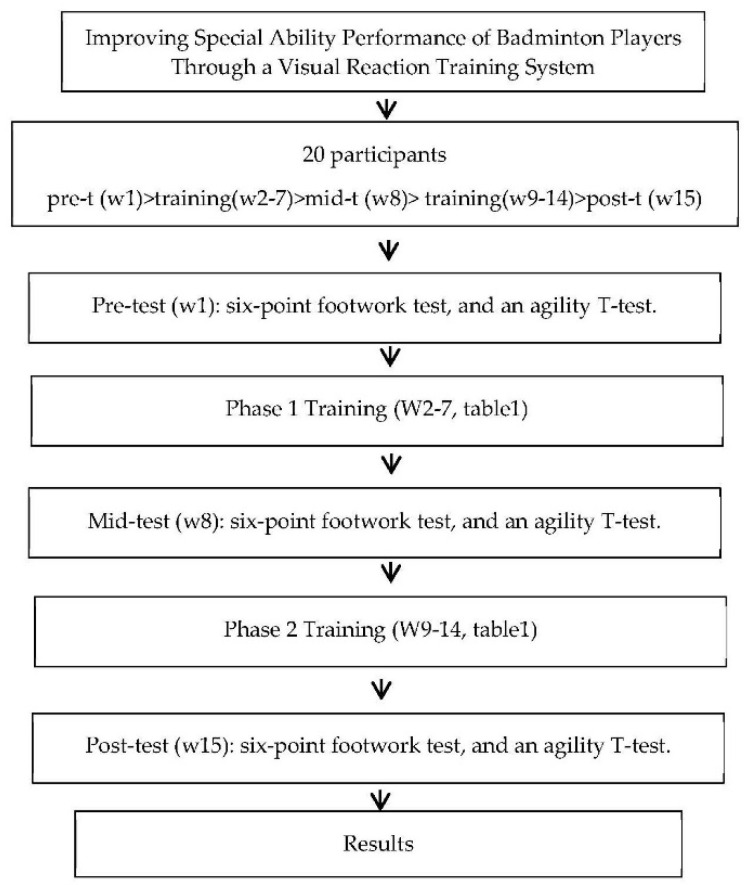
Research procedures.

Prior to the commencement of the study intervention, the purpose, methods, and procedures of the present study were explained to the participants. They then underwent a trial VRTS-based training session that enabled them to adapt to the study environment and familiarize themselves with the training equipment and methods, and the instruments were carefully inspected to ensure that they could record data. Before the formal training intervention, participants practiced familiarization with VTRS, and a rest period of at least 48 h was implemented between the end of the exercise and the start of the formal experiment to prevent participant fatigue and potential build-up. Additionally, the study was conducted for a total of 15 weeks, with participants completing the training period pretest (week 1), Phase 1 (weeks 2–7), and Phase 2 (weeks 9–14), and posttest (week 15). The pretest, midtest, and posttest were evaluated twice, and the best score was used as the experimental data (Figure 1).

**Figure 2:**
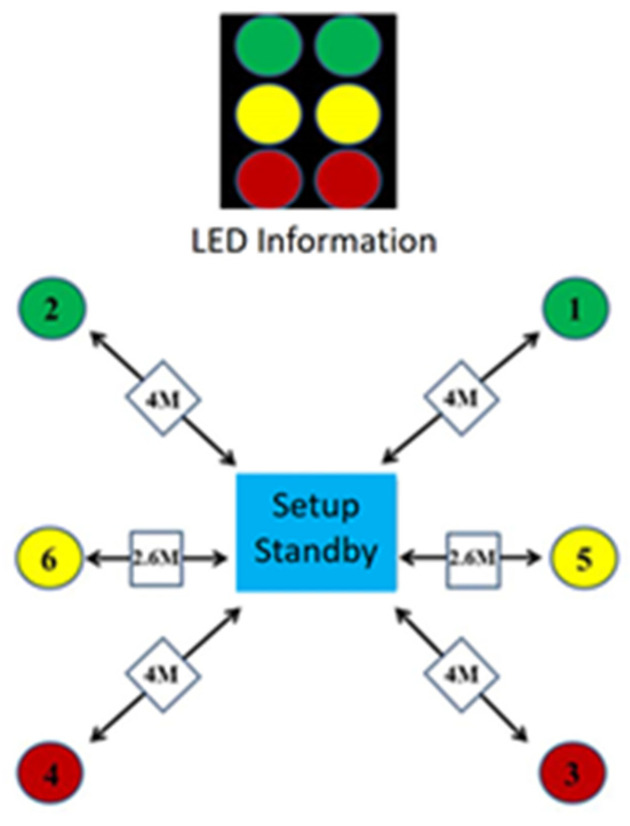
Six-point footwork test venue layout.

For the pretest, midtest, and posttest of the present study, the 20 participants underwent a fixed-light-mode six-point footwork test, a random-light-mode six-point footwork test, and an agility *t*-test. The test procedures were as follows. A visual stimulus light signal panel that indicates movement direction was placed in front of the court near the center of the net. Optical sensors were installed on the left and right sides of the front court and rear court (4 m from the central position) and on the left and right sides of the midcourt (2.6 m from the central position). Each participant moved quickly from the central position (30 cm × 30 cm) according to the direction indicated by the indicator light. Figure 2 illustrates the six-point footwork test venue layout. However, the lights indicated movement directions through two modes. In the fixed mode, the indicator lights lighted up in a specific order (i.e., Corners 1, 2, 3, 4, 5, and 6), and the participants performed the footwork movement test in accordance with the light indications. In the random mode, the LED lights lighted up in a random order to simulate a game situation.

**Figure 3:**
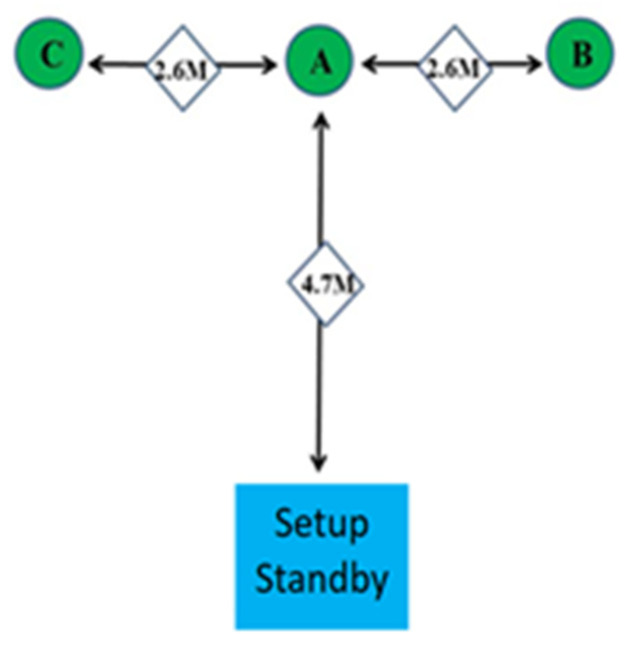
Agility *t*-test venue layout.

A participant performed the agility *t*-test by running straight from the starting position to Point A (4.7 m), moving laterally from Point A to Point B (2.6 m), moving laterally from Point B (pass Point A) to Point C (the distance between Points A and C = 2.6 m), moving laterally from Point C to Point A, and then running back to the starting position. The entire sequence from the starting position was as follows: starting position → A → B → A → C → A → starting position. The total distance covered was 19.8 m. Figure 3 illustrates the agility *t*-test venue layout.

Prior to the pretest, midtest, and posttest as well as each training session, each player was asked to perform static stretching of a specific intensity followed by a dynamic warm up to ensure that they effectively warmed up their neuromuscular system and to minimize the risk of neuromuscular injury. The present study developed a training schedule by revising the training schedule used by Kuo et al. [3]. The content of the agility training for the footwork movements is presented in Table 1.

## 4. Statistical Analysis

In the present study, a VRTS-based training intervention was conducted to enhance the agility of male high school badminton players. The system tested the players’ agility and analyzed variabilities in their reaction time, action time, and the time taken to perform movements; the outcomes of the intervention were also examined. Data from the pretest, midtest, and posttest were collected using a computer, and a difference analysis was conducted using the SPSS 20.0 statistical software package (Version 20.0; SPSS, Chicago, IL, USA). The reaction and action time data collected during the fixed- and random-light-mode six-point footwork tests and the agility *t*-test were evaluated through a one-way repeated-measures analysis of variance (ANOVA). Training outcomes were assessed by performing a Bonferroni post hoc test, and the significance level *α* for the present study was 0.05.

## 5. Results

### 5.1. Differences in Fixed-Light-Mode Six-Point Footwork Test Results

Significant differences in the reaction times recorded during the fixed-light-mode six-point footwork test were observed for all six positions at 12 weeks after the start of the VRTS-based training (Corner 1, F = 93.298, *p* ≤ 0.01; Corner 2, F = 62.714, *p* ≤ 0.01; Corner 3, F = 38.633, *p* ≤ 0.01; Corner 4, F = 123.514, *p* ≤ 0.01; Corner 5, F = 317.821, *p* ≤ 0.01; Corner 6, F = 33.673, *p* ≤ 0.01). Additionally, the post hoc test results pertaining to the reaction times for the six positions indicated a significantly superior performance for the midtest and posttest compared to the pretest. Table 2 presents the reaction time results for the fixed-light six-point footwork test with respect to these positions.

Significant differences in the total movement times recorded during the fixed-light-mode six-point footwork test were observed for all six positions (Corner 1, F = 38.117, *p* ≤ 0.01; Corner 2, F = 56.915, *p* ≤ 0.01; Corner 3, F = 8.620, *p* = 0.035; Corner 4, F = 26.625, *p* ≤ 0.01; Corner 5, F = 11.181, *p* = 0.05; Corner 6, F = 14.628, *p* = 0.01). The post hoc test results pertaining to the total action times at Corners 1, 2, 4, and 6 indicated a significantly superior performance for the midtest and posttest compared to the pretest; by contrast, a superior performance was achieved at Corner 3 for the midtest compared to the pretest and at Corner 5 for the posttest compared to the pretest. Table 3 presents the total action time results obtained through the fixed-light six-point footwork test.

### 5.2. Differences in Random-Light-Mode Six-Point Footwork Test Results

Significant differences in the reaction times recorded during the random-light-mode six-point footwork test were observed for the following positions: Corner 1, F = 3.729, *p* = 0.047; Corner 2, F = 4.296, *p* = 0.043; Corner 3, F = 8.997, *p* = 0.001; Corner 4, F = 9.386, *p* = 0.001; Corner 5, F = 4.771, *p* = 0.021. Additionally, the post hoc test results indicated that the random-light-mode reaction times recorded at Corners 2, 3, and 5 were significantly superior for the posttest compared to the pretest. For Corner 4, a significantly superior performance was obtained for the midtest and posttest compared to the pretest. Table 2 presents the results of the random-light-mode six-point footwork test.

Significant differences in the total action times recorded during the random-light-mode six-point footwork tests were observed for all six positions (Corner 1, F = 7.828, *p* = 0.003; Corner 2, F = 21.033, *p* ≤ 0.01; Corner 3, F = 4.389, *p* = 0.036; Corner 4, F = 13.995, *p* ≤ 0.01; Corner 5, F = 13.427, *p* ≤ 0.01; Corner 6, F = 20.016, *p* ≤ 0.01). The post hoc test results for action time indicated a significantly superior performance at Corners 2, 4, 5, and 6 for the midtest and posttest compared to the pretest. The post hoc test also revealed a superior performance at Corner 1 for the posttest compared to the pretest. Table 3 presents the total action time results for the random-light-mode six-point footwork test.

### 5.3. Differences in Agility t-Test Results

The results of the one-way repeated measures ANOVA revealed significant differences in the total movement times obtained through the fixed-light-mode test (F = 29.469, *p* ≤ 0.01), random-light-mode test (F = 22.381, *p* ≤ 0.01), and agility *t*-test (F = 48.229, *p* ≤ 0.01). The Bonferroni post hoc test indicated a significantly superior performance for total movement time during the midtest and posttest compared to during the pretest. Table 4 presents the results of the agility tests.

## 6. Discussion

The self-developed VRTS tested in the present study was used to develop training schedules. The system provided data feedback that contributed to improving training outcomes. An electronic device was developed to facilitate the training of an essential badminton-specific skill (i.e., footwork during quick attacking and defensive movements) to improve the agility of players.

The participants underwent a 12-week agility training intervention that was based on the VRTS and comprised two types of badminton-specific footwork movements (i.e., six-point footwork and T-footwork). The results reveal significant differences in the reaction and action times recorded for the fixed-light-mode six-point footwork test. For the random-light-mode six-point footwork test, only the reaction times recorded at Corner 6 were not significantly different because the Corner 6 direction was to the left of the players, and in badminton footwork, only one step was required to complete the action at Corner 6. Therefore, the time taken was short, and the variability in action time was small. By contrast, the variability in reaction and action times was greater at other positions, and the results revealed significant differences when changes occurred in these positions. Furthermore, the results indicated that the VRTS-based training, which focused on the agility of players during movements, enabled them to improve their time performance from the pretest to the midtest. Additionally, their performance in the posttest was superior to their performance in the pretest and midtest, indicating that the VRTS-based training of footwork during movements allows high school players to effectively improve their reaction and action time performance.

The training of badminton-specific footwork is a major component of agility training. Additional information was obtained through scientific quantitative results regarding the training, and it served as further feedback for coaches and players. In contrast to inaccurate measurement methods (e.g., using stopwatches), the VRTS was designed based on exercise science, and it is light, portable, and straightforward to set up. When used with a computer and a sensor, the VRTS can precisely record various types of information regarding the agility of players; in addition, laboratory space constraints and other limitations are addressed through the system’s accurate quantitative measurements such that the agile reactions of players can be monitored during training. The experimental results indicated that the system can effectively contribute to monitoring, recording, and analytical tasks during training and collect relevant quantitative data. The quantitative data that the system delivers to coaches and players can further facilitate the planning of subsequent training.

Studies have compared badminton players with varying skill levels by conducting badminton-specific speed and sprint tests. Their results indicated that badminton-specific speed tests are superior to general sprint tests for assessing agility performance because of their closer correlation with athletic performance in the context of badminton; precise training allows for effective player selection and training course design [26]. The findings of the present study indicated that both the reaction times and the action times of the participants adequately improved following VRTS-based training. The action time results of the participants revealed superior posttest results for the backhand side relative to the forehand side. For random-light reaction time, the results revealed a shorter reaction time for the forehand side than for the backhand side. The players moved by shifting their center of gravity after receiving stimulating information; this principle conforms with the theory of perception and decision-making in agility [2,18,19], indicating a crucial association between agility and specific athletic performance. Similarly, the results of the present study revealed significant improvements in the reaction time performance of its participants.

Studies have also indicated that the state of a player’s central nervous system can be improved by enhancing their agility during routine training to facilitate their processing and decision-making regarding stimulating information, and their reaction speed also increased [27]. During the VRTS-based training of agility for footwork movements, indicator lights produced rapid and changing stimulating information that served as cues for the participants; this method effectively trained the players to reduce their reaction time, thereby improving their agility.

Ooi et al. tested the agility of elite and sub-elite Malaysian badminton players and reported the absence of significant differences between the two groups [28]. The training results obtained through tests conducted in other studies were similar because these tests focused only on fixed and specific directions. Through the implementation of a tournament simulation mode, the VRTS-based training provides increased variety. The random-light tournament mode stimulated the participants to react agilely, and significant differences were observed between pretest results and midtest and posttest results, indicating that the system substantially contributed to the training of the participants and improved their performance.

Studies have used the six-point position model to test the agility of players in various areas. The results indicated that, although agility is an ability required in various sports events, the six-point position agility test is more suitable for assessing the agility of badminton players than for assessing the agility of players of other sports; this is because badminton players perform agile movements within a smaller space relative to players of other sports. Moreover, sports science researchers should focus on simultaneous training and testing of badminton-specific abilities to enable badminton players to apply their skills and improve their athletic performance during tournaments more efficiently [29]. Based on the findings, the six-point footwork test is recommended for assessing the movements of badminton players during training sessions and tournaments. Based on six-point footwork tests that are conducted in fixed and random light modes, the VRTS measures a player’s reaction time and action time. It provides effective feedback that further enhances the training and strategy of players with respect to their footwork movements, thereby enhancing their agility while reinforcing their shifting and running within a badminton court.

The results of a Taiwan-based retrospective study on badminton footwork suggested that the routine training of badminton players should involve visual stimulation training followed by motor control training. This training method can improve the perceptual and decision-making abilities of badminton players [30]. Accordingly, the present study obtained test data through simulated tournament scenarios and examined the changes in direction on the left and right sides of the forecourt, midcourt, and rear court. The ability to change direction was tested, participants were randomly provided with stimulating information, and their ability to move in various directions was measured. The training module used in the present study corresponds to theories regarding the definition of agility, and it covers information reception, reactions and actions, the ability to change direction, and other mechanisms. Another study recruited 43 badminton players (29 men and 14 women) and conducted an agility test that was similar to the one conducted in the present study. Its results indicated that specific tests are necessary for badminton players. Meanwhile, a wide range of data can be obtained through specific quantitative data-based tests, and athletic performance can be further improved with the aid of technology [31].

After a badminton player executes a shot, they must return to the center of the court and react to their opponent’s returning shot by performing rapid six-point footwork or T-footwork movements to move to the forecourt, the rear court, or the sides of the court. Therefore, by determining the correct direction and rapidly moving to the correct position through the execution of appropriate footwork movements, players can correctly and effectively apply their skills and return shots [4]. In the present study, significant differences in agility *t*-test results were observed, indicating that the VRTS-based training can adequately improve the ability of badminton players to move rapidly in straight and lateral directions, enabling them to perform highly efficient shots.

## 7. Conclusions

The present study used a VRTS to train 20 male high school badminton players and demonstrated that the system substantially improved their reaction and action time performance, thereby enhancing their abilities and agility. The VRTS can provide quantitative data on badminton-specific training. A wide range of information is made available to coaches and players for testing, enabling them to facilitate specific training precisely and effectively and, thus, improve training outcomes and athletic performance.

During the training conducted for the present experiment, the test data pertaining to specific abilities and agility were collected only from male high school badminton players who received regular training; no VRTS-based test was conducted to collect data from female players. Future studies should recruit female players and players of varying technical levels, incorporate hitting actions, or simulate various tournament scenarios and include other evaluation indicators (e.g., electromyography, and photography-based motion analysis.). As an electronic training system, the VRTS is arranged according to different training methods and intensities, and research has proved that it can effectively improve an athlete’s agility and mobility, and effectively improve training and performance in the context of badminton (or other sports). Therefore, the VTRS is a scientific instrument with both training function and detection and evaluation ability.

## Figures and Tables

**Table 1:** Training content.

Week 1	Pretest	T-Footwork Movement: 2n × 2N Six-Point Footwork 2C × 2n × 1N & 2R × 2n × 1N
Week/Item and assignment	Fixed direction	Random direction
Weeks 2 and 3	2C × 2n × 2N	2C × 3n × 1N
	Rest interval between sets = 30 s	Rest interval between sets = 30 s
Weeks 4 and 5	2C × 2n × 2N	2C × 3n × 2N
	Rest interval between sets = 30 s	Rest interval between sets = 30 s
Weeks 6 and 7	2C × 2n × 2N	2C × 3n × 2N
	Rest interval between sets = 20 s	Rest interval between sets = 20 s
Week 8	Midtest	T-footwork movement: 2n × 2N Six-point footwork: 2C × 2n × 1N & 2R × 2n × 1N
Weeks 9 and 10	2C × 3n × 2N	2C × 3n × 2N
	Rest interval between sets = 20 s	Rest interval between sets = 20 s
Weeks 11 and 12	2C × 3n × 2N	3C × 2n × 2N
	Rest interval between sets = 20 s	Rest interval between sets = 20 s
Weeks 13 and 14	2C × 3n × 2N	1C × 5n × 2N
	Rest interval between sets = 20 s	Rest interval between sets = 10 s
Week 15	Posttest	T-footwork movement: 2n × 2N Six-point footwork: 2C × 2n × 1N & 2R × 2n × 1N

C = completion of fixed-mode six-point positions; R = completion of random-mode six-point positions; n = repetitions per set; N = number of sets.

**Table 2:** Reaction times for footwork movements in six-point positions in fixed and random modes.

	Pretest	Midtest	Posttest	F	Power
Fixed-mode six-point reaction time_Corner 1	0.715 ± 0.118	0.191 ± 0.072	0.209 ± 0.081	93.298 **	1.000
Fixed-mode six-point reaction time_Corner 2	0.739 ± 0.332	0.201 ± 0.065	0.139 ± 0.067	62.714 **	1.000
Fixed-mode six-point reaction time_Corner 3	0.679 ± 0.385	0.112 ± 0.074	0.151 ± 0.102	38.633 **	1.000
Fixed-mode six-point reaction time_Corner 4	0.758 ± 0.221	0.167 ± 0.238	0.131 ± 0.066	123.514 **	1.000
Fixed-mode six-point reaction time_Corner 5	0.684 ± 0.115	0.146 ± 0.083	0.119 ± 0.060	317.821 **	1.000
Fixed-mode six-point reaction time_Corner 6	0.686 ± 0.201	0.174 ± 0.087	0.148 ± 0.215	33.673 **	1.000
Random-mode six-point reaction time_Corner 1	0.778 ± 0.174	0.617 ± 0.452	0.636 ± 0.098	3.729 *	0.593
Random-mode six-point reaction time_Corner 2	0.952 ± 0.166	0.729 ± 0.352	0.659 ± 0.318	4.296 *	0.694
Random-mode six-point reaction time_Corner 3	0.901 ± 0.211	0.716 ± 0.179	0.563 ± 0.149	8.997 *	0.962
Random-mode six-point reaction time_Corner 4	0.981 ± 0.167	0.686 ± 0.277	0.665 ± 0.273	9.386 *	0.961
Random-mode six-point reaction time_Corner 5	0.830 ± 0.124	0.755 ± 0.178	0.659 ± 0.175	4.771 *	0.724
Random-mode six-point reaction time_Corner 6	0.858 ± 0.224	0.863 ± 0.253	0.769 ± 0.162	2.291	0.319

*: *p* < 0.05; **: *p* < 0.01.

**Table 3:** Time taken for footwork movements in six-point positions in fixed and random modes.

	Pretest	Midtest	Posttest	F	Power
Time for fixed-mode six-point footwork_Corner 1	3.485 ± 0.582	2.296 ± 0.335	2.279 ± 0.271	38.117 **	1.000
Time for fixed-mode six-point footwork_Corner 2	3.318 ± 0.132	2.654 ± 0.354	2.401 ± 0.108	56.915 **	1.000
Time for fixed-mode six-point footwork_Corner 3	3.701 ± 0.718	2.699 ± 0.261	3.127 ± 0.586	8.620 **	0.798
Time for fixed-mode six-point footwork_Corner 4	3.317 ± 0.294	2.892 ± 0.419	2.662 ± 0.245	26.625 **	1.000
Time for fixed-mode six-point footwork_Corner 5	2.568 ± 0.553	1.916 ± 0.248	1.799 ± 0.239	11.181 *	0.889
Time for fixed-mode six-point footwork_Corner 6	2.397 ± 0.571	1.701 ± 0.295	1.875 ± 0.166	14.628 **	0.993
Time for random-mode six-point footwork_Corner 1	3.778 ± 0.832	2.921 ± 0.619	2.801 ± 0.408	7.828 *	0.919
Time for random-mode six-point footwork_Corner 2	3.117 ± 0.199	2.729 ± 0.357	2.457 ± 0.112	21.033 **	1.000
Time for random-mode six-point footwork_Corner 3	3.369 ± 0.352	3.201 ± 0.292	3.152 ± 0.284	4.389 *	0.708
Time for random-mode six-point footwork_Corner 4	3.355 ± 0.296	2.822 ± 0.486	2.708 ± 0.321	13.995 **	0.996
Time for random-mode six-point footwork_Corner 5	2.509 ± 0.338	2.110 ± 0.371	1.993 ± 0.237	13.427 **	0.993
Time for random-mode six-point footwork_Corner 6	2.415 ± 0.372	1.836 ± 0.235	1.659 ± 0.226	20.016 **	1.000

*: *p* < 0.05; **: *p* < 0.01.

**Table 4:** Movement time obtained through agility test.

	Pretest	Midtest	Posttest	F	Power
Total movement time for fixed-mode six-point footwork	19.010 ± 2.1721	14.208 ± 1.328	13.989 ± 1.436	29.469 **	0.999
Total movement time for random-mode six-point footwork	18.162 ± 1.917	16.663 ± 1.277	14.927 ± 1.259	22.381 **	1.000
Total movement time for T agility running	17.558 ± 1.443	14.594 ± 1.315	14.356 ± 1.108	48.229 **	1.000

**: *p* < 0.01.

## Data Availability

Not applicable.
